# Data collected in a citizen scientist study uncover a new species record of *Phoxinus* minnow for Austria

**DOI:** 10.1007/s10661-026-15168-6

**Published:** 2026-03-15

**Authors:** Min J. Chai, Nina G. Bogutskaya, Susanne Reier, Rok Friedrich, Hans Rund, Sabine Wanzenböck, Josef Wanzenböck, Florian Glaser, Silvia Marcante, Ilka Prowatke, Michael Jung, Ernst Mikschi, Anja Palandačić

**Affiliations:** 1 First Zoological Department, Vienna Museum of Natural History, Burgring 7, 1010 Vienna, Austria; 2 BIOTA J d.o.o., Dolga Gora 2, 3232 Ponikva, Slovenia; 3 Research Department for Limnology Mondsee, University of Innsbruck, Mondseestraße 9, 5310 Mondsee, Austria; 4 Technical Office for Biology Dr. Florian Glaser, Walderstraße 32, Absam, 6067, Austria; 5 Verein Waldorfpädagogik, Jahnstraße 5, 6020 Innsbruck, Austria; 6 Öffentliches Gymnasium, Franziskaner Hall, Kathreinstraße 6, 6060 Hall in Tirol, Austria; 7 Technical Office for Applied Aquatic Ecology, Fisheries Management, Cultural Engineering and Water Management, Marktstraße 35, 4090 Engelhartszell, Austria; 8 Department of Biology, Biotechnical Faculty, University of Ljubljana, Večna pot 111, 1000 Ljubljana, Slovenia

**Keywords:** Fish introductions, Live-bait fishing, Admixed populations, DNA barcoding, Environmental DNA

## Abstract

Freshwaters are among the most vulnerable ecosystems, yet the scarcity of biodiversity assessments prevents the detection of changes incurred by neobiota. Minnows of the genus *Phoxinus* were long thought to be represented by a single species in Eurasia, the common minnow *P. phoxinus*, but the genus now includes more than 25 valid species. However, their distributions do not follow drainage boundaries, there are known cases of human translocations, and morphological species assignation is difficult due to intra- and interpopulation phenotypic diversity. Hence, the species were delimited and are now determined mostly using molecular methods. In Austria, recent studies have identified at least four different species of *Phoxinus*, three of which are considered native and one introduced. However, more data were needed; thus, extensive collecting and DNA barcoding of minnow populations was undertaken with the help of recreational fishers, school pupils, and field biologists. DNA barcodes of museum specimens and environmental DNA collected from water samples were also included. Altogether, the genetic lineage of 258 new *Phoxinus* specimens was determined. The results confirmed the distribution of *P. marsilii* in eastern Austria, *P. lumaireul* in southern Austria and *P. csikii* in central and western Austria. Additional populations of the introduced *P. phoxinus* were identified. Most importantly, a new species record for Austria, *P.* cf. *morella,* was discovered, yet it is unclear whether its distribution in Austria is natural. This study also confirmed the potential of citizen science for biodiversity monitoring, with the number of specimens analyzed increasing fourfold in just two years.

**Supplementary Information:**

The online version contains supplementary material available at 10.1007/s10661-026-15168-6.

## Introduction

Freshwaters are among the most endangered ecosystems, with invasive/introduced species being one of the main threats (Linders et al., 2019). The problem is exacerbated by a lack of data, as biodiversity assessments for many freshwater ecosystems are incomplete, sparse, or absent. In the case of *Phoxinus* minnows (Leuciscidae), it was believed for over a century that a single species, the Eurasian minnow *P. phoxinus* (Linnaeus, 1758), was distributed throughout Eurasia. However, starting with keystone studies based on morphological (Bogutskaya and Naseka, 2004; Kottelat, 2007) and molecular data (Geiger et al., 2014; Palandačić et al., 2015), a much higher biodiversity within the genus has been revealed over the past 20 years. To date, the Catalogue of Fishes (Fricke et al., 2025 and references therein) formally recognizes up to 28 valid species of *Phoxinus*, but at least five of these require further taxonomic validation (see, for example, the comments in Artaev et al. (2024)). Additionally, several currently unassigned, mitochondrial (mt) lineages remain within the genus *Phoxinus* (Palandačić et al., 2020, 2022). The distribution area of *P. phoxinus* has shrunk to the Rhine, Seine, and Meuse drainages in northern Germany, France, and the Netherlands (Denys et al., 2020; Palandačić et al., 2022; Sternberg et al., 2025). The hidden biodiversity in the *Phoxinus* genus has largely been revealed by molecular methods such as DNA barcoding, since morphological delineation and identification of species remain challenging due to inter- and intrapopulation phenotypic diversity (Bogutskaya et al., 2020; Palandačić et al., 2024a, 2024b).

*Phoxinus* minnows have limited commercial value, i.e., they are not used for consumption; however, they are introduced as fry for predatory fishes (Corral‐Lou et al., 2019; De Santis et al., 2021; Denys et al., 2020; Palandačić et al., 2020), used for live-bait fishing (Corral‐Lou et al., 2019; Garcia-Raventós et al., 2020; Museth et al., 2007), and kept as ornamental fish in ponds (Maceda-Veiga et al., 2013). These intentional introductions as prey for predatory fish or accidental introductions with other fry have resulted in local populations hybridizing with introduced populations in Italy, Spain, France, Germany, and Austria (Corral‐Lou et al., 2019; De Santis et al., 2021; Denys et al., 2020; Palandačić et al., 2020; Sternberg et al., 2025). Furthermore, live-bait fishing has resulted in the introduction of *Phoxinus* to Norway, the Spanish Pyrenees, the Douro drainage basin in Portugal, and Corsica (Corral‐Lou et al., 2019; Esposito et al., 2024; Garcia-Raventós et al., 2020; Museth et al., 2007). Due to the numerous introductions and translocations, *Phoxinus* minnow species distributions are complex, particularly as they also reflect palaeohydrological conditions (Palandačić et al., 2020; Reier et al., 2022, 2025). To better understand the current distribution of *Phoxinus* species and their distributions prior to the large-scale introduction of fishes across Europe, which, according to Crivelli (1995), occurred within the last 70 years, museum specimens up to 200 years old were successfully used (Palandačić et al., 2020).

Many studies have identified habitat degradation and invasive species as the primary causes of biodiversity loss (e.g., Clavero & García-Berthou, 2006; Didham et al., 2007; Hermoso et al., 2011). Similarly, genetic introgression caused by the introduction of non-native congeners is not the only threat to *Phoxinus* minnows; habitat loss and climate change also pose a significant risk. As small, schooling fishes that prefer cool, oxygen-rich water (Frost, 1943), they are susceptible to rising water temperatures (Závorka et al., 2020). As gravel spawners, *Phoxinus* require silt-free gravel through which oxygenated water can percolate between the grains. Thus, sediment accumulation (i.e., siltation) due to agriculture, forest exploitation, and hydropower plants may affect their reproductive success (Graf et al., 2016; Hauer et al., 2013; Krapesch et al., 2023). Personal communication with fishers in Austria also suggests that *Phoxinus* minnows are affected by the dewatering of spawning grounds due to fluctuations in water levels caused by hydropeaking and wave action by ship traffic, as is the case for many other aquatic species (Bipa et al., 2024; Nagrodski et al., 2012). *Phoxinus* minnows are also susceptible to noise pollution (Currie et al., 2021). Finally, competition for trophic resources and prey between native *Phoxinus* populations and their non-native congeners (Britton, 2023; Cruz et al., 2022) may also affect them, as may the transmission of pathogens and parasites from the introduced species (Esposito et al., 2024). Thus, the conservation of *Phoxinu*s minnows is a complex, multilevel process. Firstly, sufficient data on the distribution of individual species should be collected. Secondly, regular monitoring should be carried out. Thirdly, if necessary, well-planned restoration projects using native populations should be implemented. Due to the difficulty of identifying species morphologically, field surveys alone are insufficient for the successful implementation of these three steps; rather, genetic analysis of *Phoxinus* minnows is necessary.

Similarly as in Europe, recent studies of *Phoxinus* in Austria have identified not one but at least four distinct species of *Phoxinus*. Of these, three species — *P. marsilii* Heckel, 1836*, P. csikii* Hankó, 1922 and *P. lumaireul* (Schinz, 1840) — are considered native, while *P. phoxinus* is an introduced species (Palandačić et al., 2020). Of the above mentioned threats to minnow populations in Austria, hybridization with non-native congeners, rising water temperatures and habitat degradation have been identified (Palandačić et al., 2017, 2020; Závorka et al., 2020). However, to protect *Phoxinus* minnows in Austria, additional data on species diversity and distribution, including that of introduced populations, were needed.

Therefore, the first aim of this study was to conduct an extensive DNA barcoding study (sensu Hebert et al. (2003)) on *Phoxinus* minnow populations across Austria. As Austrian legislation concerning fish collection and sampling is extremely complicated, e.g., each of the nine federal states has its own requirements and requires a separate permit, the sampling strategy relied heavily on recreational fishers and fishing societies, who have the appropriate permits. Pupils supported by fishers and scientists were also included in the collection of data. Finally, biologists performing freshwater field surveys were also involved. The second aim of the study was therefore to assess the feasibility of engaging fishers as citizen scientists to collect nationwide data for fish biodiversity monitoring. To expand the dataset and enable comparison of past and present patterns of species diversity and distribution, museum specimens — some of which are over 200 years old — were also used. Finally, environmental DNA (eDNA) from water samples was analyzed and added to the dataset. Despite its limitations, eDNA metabarcoding of fish is an efficient monitoring tool and a valuable alternative to capture-based methods (see, e.g., Curto et al., 2025; Sahu et al., 2025, and references therein). Furthermore, collecting and analyzing water does not require permits for sampling and handling fish, and is thus a promising method for use in nationwide monitoring programmes aided by citizen scientists. The third aim of this study was therefore to test whether MiFish primers, which primarily amplify a short fragment of the mitochondrial 12S RNA gene for fish species diversity assessments (Hänfling et al., 2016; Rund et al., 2025), are sufficient for detecting inter- and intraspecific genetic lineages in Austrian *Phoxinus* minnows.

## Materials and methods

### Specimens, fin clips, and mucus swabs collection and processing

Specimens were collected in the frame of a citizen science project “Biodiversity of minnows in Austria,” funded by Austria’s Agency for Education and Internationalisation (OeAD) of the Federal Ministry of Women, Science and Research as part of the “Sparkling Science 2.0” programme (https://elritzen.at/; in German) between the years (Palandačić, et al., 2022) and 2025. They were caught under the permission of the concerned state, federal, or private agencies and institutions and in accordance with the Austrian state laws on fisheries. Specimens were collected in different water bodies (streams, rivers, lakes, private and state ponds) across Austria by recreational fishers, pupils, and as part of annual monitoring surveys by field biologists. Specimens were caught by traps, hand nets, or electrofishing. Altogether, tissue samples of 346 specimens were collected and sent back to the Natural History Museum Vienna (NHMW); 208 were fin clips and 138 were (mucus) swabs. Swabs used are 4N6FLOQSWAB Plastic Tubes from Thermo Fisher Scientific. Fish are sampled by swabbing them laterally a few times from the head to the tail (instructions were prepared for the user and are available at https://elritzen.at/wp-content/uploads/2023/02/Beprobung-mit-Tupfer.pdf). Additionally, 67 whole fish were sent in. All this material (*N* = 413; Supplementary Table S1) is designated herein as fresh material.

Forty museum specimens were added to the dataset (designated herein as historical material). The term historical specimens is applied here to museum samples collected prior to 2000. These specimens were mostly stored in ethanol, while some were stored in formalin (Suppl. Table S1). In fishes, conservation methods can be distinguished, as alcohol-fixed specimens possess white eyes, while formalin-fixed fishes have clear black eyes (De Bruyn et al., 2011). Of the whole fresh and museum fish specimens, branchial arches from the right side were sampled and used for DNA extraction (Suppl. Table S1). Finally, 43 sequences from Austria from previous studies were included in the dataset (for the GenBank numbers see Suppl. Table S1).

The material was distributed unevenly in terms of the abundance of specimens per sampling site/body of water. Therefore, in areas where a large number of specimens were collected, DNA was extracted from only a small number of individuals, typically five to seven. DNA extraction from fresh material (clips, swabs and branchial tissue) was performed using the QIAmp DNeasy Blood and Tissue Kit (Qiagen, Hilden, Germany) following manufacturer’s protocol. For the historical material, DNA extraction from branchial tissue was conducted using the QIAmp DNeasy Blood and Tissue Micro Kit (Qiagen, Germany), with the addition of 40 µl Dithiothreitol (DTT) to the lysis buffer.

After the DNA extraction of the fresh material, polymerase chain reaction (PCR) was used to amplify the 651 base pairs (bp) long barcoding region of mitochondrial (mt) cytochrome c oxidase subunit I (COI), as described in Palandačić et al. (2017). In contrast, for the historical material, the COI gene was amplified in shorter fragments, varying in length from 150 to 263 bp and subsequently concatenated into a single sequence (for protocol and primers see Palandačić et al. (2017)). PCR products were cleaned up with QIAquick PCR Purification Kit (Qiagen, Hilden, Germany) and either sequenced in one direction (fresh material) or sequenced bidirectionally (historical material) at Microsynth (Balgach, Switzerland) using the PCR primers.

After sequencing, chromatograms were checked and raw sequences were edited, assembled and aligned using MEGA 6.0 (Tamura et al., 2013). The following analyses were performed in two steps. In the first step, respective mt genetic lineages of all newly sequenced DNA samples were assigned by neighbor-joining (NJ) phylogenetic tree reconstruction in MEGA 6.0 using maximum composite likelihood method, 500 bootstraps and, as reference, COI sequences from other studies (see Suppl. Table S1 for GenBank numbers). The alignment had no missing data or ambiguities. In the second step, phylogenetic reconstruction and haplotype network construction were conducted with all newly acquired sequences and the reference sequences of corresponding species/genetic lineages which are present in Austria (see Suppl. Table S1 for GenBank numbers). These analyses were employed to assess the haplotype distribution of native species/genetic lineages and to identify the possible introductory sources/routes of introduced species/lineages. As shown previously (e.g., Palandačić et al., 2020), the barcoding of CO1 fragment is not sufficient to resolve deep phylogenies in the *Phoxinus* genus, and the focus of this study was primarily species/clades identification rather than reconstruction of phylogenetic relationships. In this case, unrooted trees are beneficial in depicting clusters of related sequences (Kinene et al., 2016). Phylogenetic inference was performed using maximum likelihood (ML) and Bayesian inference (BI) approaches implemented in the PhyloSuite v.1.2.2 software (Zhang et al., 2020). The alignment was collapsed with DNAcollapser implemented in FaBox (Villesen, 2007) and had no missing data. The alignment was then realigned using MAFFT 7 (Katoh & Standley, 2013) and the most appropriate evolutionary model was determined using PartitionFinder2 (Lanfear et al., 2016). Tree search was performed with IQtree (Nguyen et al., 2015) and MrBayes v.3.2.6 (Ronquist et al., 2012), all implemented in PhyloSuite. Finally, using the same dataset (sequences of Austrian samples and corresponding genetic lineages and species), a median-joining haplotype network implemented in PopART v1.7 software (Leigh & Bryant, 2015) was produced using default settings.

### eDNA water sample collection and processing

All eDNA samples were collected within other projects (Suppl. Table 1) but were re-analyzed for *Phoxinus* DNA within this study. Each sample taken within the CLAIMES project (sampling was conducted in September of 2019 and 2020) consisted of 2 L (L) of lake water and was collected directly from the shore of the respective lake using previously sterilized (24 h incubation in 10% hydrogen peroxide (H_2_O_2_)) 2.5 L plastic containers. All the other eDNA samples consisted of 5 L of lake or river water and were either collected in September 2019 or in October 2020 and 2021. Water samples obtained from lakes (excluding the CLAIMES samples) were collected at specified coordinates at different depths using a Schindler-Patalas sampler. After each deployment, the sampler was emptied into a sterilized 5 L plastic container and subjected to decontamination measures (as described in Rund et al. (2025)). Samples taken in rivers were collected at the surface, either at the riverbanks or the middle of the river, using sterilized 5 L plastic containers. Each sampling site was sampled once, and no biological replicates were collected. After sampling, the water samples were transported to the Research Department for Limnology, Mondsee at ambient temperatures but protected from UV-radiation in order to avoid DNA degradation. All water samples were then filtered through ⌀47 mm GFC filter discs (Whatman 1822-047, approx. pore size 1.2 μm) using a vacuum filtration manifold that allowed parallel filtration of six samples. After each round of filtration, the filter cups and holders were subjected to an incubation period of one hour in a 10% H_2_O_2_ solution. Six samples were filtered simultaneously, comprising five eDNA samples and one filtration blank (5 L of MiliQ water), to detect potential cross-contamination during filtration. These blanks were treated in the same way as the other samples with regard to DNA extraction and subsequent analysis. Post-filtration, the filters were stored at −20 °C until DNA extraction.

Prior to DNA extraction, all work surfaces and equipment (e.g. forceps) were decontaminated using a 10% H_2_O_2_ solution and 70% ethanol (EtOH). DNA was extracted from the GFC filters using the DNeasy PowerWater Kit (Qiagen, Hilden, Germany) as described in the manufacturer’s protocol. The extracted DNA was eluted in 100 µl of EB buffer solution (10 mM Tris-Cl, pH 8.5). Following extraction, the total DNA concentration was quantified using the Qubit 3.0 fluorometer and the Broad-Range (BR) Assay Kit (Invitrogen, Thermo Fisher Scientific, Waltham, USA) following the manufacturer’s protocol. All blanks were found to contain DNA concentrations below the limit of detection (below 1 ng/μl using the BR Assay Kit). In addition, randomly selected blanks were subjected to PCR amplification and sequencing. However, no successful DNA amplification was detected, indicating that no contamination occurred during filtration. The DNA extracts were stored frozen at −20 °C until sequencing.

Before sequencing, all DNA extracts were analyzed for the presence of fish DNA with quantitative PCR (Teleo primer pair; Valentini et al., (2016)), in order to identify which water samples were promising for follow-up analysis. Metabarcoding was then performed at the Austrian Agency for Food Safety (AGES) using the Illumina MiSeq platform, with library preparation and sequencing performed according to Rund et al. (2024). A 176 bp fragment of the 12S rRNA gene was amplified and sequenced using the MiFish-U primers (Miya et al., 2015). Sequencing data were processed and analyzed using the QIIME 2 pipeline (Bolyen et al., 2019; version 2020.8). Cutadapt (Martin, 2011) was used to trim primers from the demultiplexed paired-end sequences; if no primer could be trimmed, the sequences were discarded. These sequences were then denoised and merged using the divisive amplicon denoising algorithm — DADA2 (Callahan et al., 2016), which enables the accurate resolution of sequence variants (ASVs), even when there is variation at the level of a single nucleotide. Due to the high number of *Phoxinus* ASV numbers, we applied a rather conservative cut-off threshold of 1%. Therefore, all ASVs representing less than 1% of the reads in each sample were excluded from the dataset to remove PCR artifacts and erroneous sequences. The ASVs were classified using a machine learning approach based on the Scikit-Learn Naive Bayes classifier (Pedregosa et al., 2011). The classifier was previously trained using a curated, in-house 12S rDNA barcode reference database (Rund et al., 2025) to enhance classification depth and confidence thresholds (Werner et al., 2012). This database contains 78 fish species (represented by 126 sequences) known to inhabit Austrian waters, as well as invasive and potentially invasive species.

### Sequencing and assembly of complete mitochondrial genomes and 12S RNA gene fragment analysis

As written in detail in the introduction, it was long believed that there is only one species of *Phoxinus* in Europe. Also, in eDNA analysis, using the short 12S mt fragment produced in metabarcoding, the *Phoxinus* genus could be identified, but there have been no studies that tested the use of metabarcoding with 12S and the MiFish-U primers for the identification of different *Phoxinus* species or further mt genetic lineages. Thus, herein, complete mt genomes of the species and lineages occurring in and around Austria were assembled. These genomes were assembled from the representatives of *P. lumaireul* — lineages 1a, 1c and 1d; *P. csikii* lineage 5b, *P. marsilii* lineage 9a and *P.* cf. *morella* lineage 11, *P. septimaniae* Kottelat, 2007 lineage 12 and *P. phoxinus* lineage 10 (lineages are coded according to Palandačić et al. (2020)), their lineages determined by COI barcoding. Subsequently, the DNA was extracted and sent for paired-end whole genome sequencing on the Illumina MiSeq platform (IGA Technology Services, Udine, Italy). The raw sequence reads were trimmed with trimgalore v0.6 (Krueger et al., 2023) using a quality threshold of 30 and a minimum length filter of 60 bp. Complete mt genomes were assembled using Geneious v 10.2.6 (http://www.geneious.com; for details see Palandačić et al. (2024b)), allowing for a sufficient coverage of ≥ 30×. First, the reads were blasted to the reference sequence of the complete mitochondrial genome of *P. phoxinus* (NCBI GenBank accession number NC_020358). In the second step, the blasted reads were aligned to the same sequence (NC_020358) and a consensus sequence was generated and annotated. Finally, the short 12S fragment, used in eDNA analyses, was extracted from the mt genomes consensus sequences and aligned with eDNA sequences produced in this study. In addition, 12S sequences available in the GenBank were added to the dataset (Suppl. Table S1). Their respective mt genetic lineages/species were assigned using NJ phylogenetic tree reconstruction in MEGA 6.0 using maximum composite likelihood and 500 bootstraps. With the same dataset, a median-joining haplotype network was constructed using PopART v1.7 software (Leigh & Bryant, 2015) with the default settings.

### Data collection of abiotic parameters and habitat features

As outlined in the introduction, *Phoxinus* minnows are susceptible to changes/loss of habitat; however, in some cases, they still thrive in highly urbanized areas (personal observation, AP). To assess the habitat characteristics where *Phoxinus* minnows were collected, participants were asked to collect not only DNA samples (from specimens, fin clips and mucus swabs), but also to fill out a standardized form (in German, available in the supplementary material and at https://elritzen.at/lehrmaterial/) describing the sampling site. The parameters included data on elevation, type of water body (stagnant/running), substrate (bigger rocks/sand/gravel/artificial), water plants (yes/no), turbidity (none/middle/strong), condition of the water body (near-natural/partially influenced/strongly influenced), bank (soil/natural stone bank/artificial bank), river embankment (steep/flat), riverbank vegetation (trees/shrubs/meadow/none), water width [m], water depth [m], water temperature, pH value, conductibility [μS/cm], catching method, date, collector and GPS coordinates. Pupils were provided with measuring gear, a VOLTCRAFT KBM-700 combi-measuring instrument for conductivity, pH value, Redox (ORP), temperature and salt. All participants were also encouraged to send photographs of the water body and sampling sites. The distribution of data by different parameters (basic descriptive statistics) was presented in Excel (Supplementary Table S2).

## Results

### Specimens, fin clips and mucus swabs collection and processing

Of the fresh material (fin clips, swabs, complete fish), a total of 413 specimens/tissues/swabs across Austria were acquired. Most of them came from Lower Austria (142), followed by Tyrol (90), Styria (47), Upper Austria (56), Vienna (24), Salzburg (16), Vorarlberg (32), and Carinthia (6), whereas from Burgenland, where nowadays *Phoxinus* minnows are only distributed in two streams at the border to Lower Austria (Wolfram et al., 2022), no samples were received. Overall, 136 specimens (33%) were collected by fishers, 100 by pupils (24%), and 177 by field biologists (43%).

The total number of DNA extracts was 264 for fresh material. For historical material, we extracted DNA from 40 specimens. PCR was successful for 233 fresh (88%) and 15 historical (37,5%) DNA samples. Of the 127 DNA samples extracted from swabs, 96 (76%) were successfully amplified by PCR. This is possibly due to the condition of the swabs — some came in moldy. The success rate for fin clips and fresh whole specimens (137 samples) was 100%. For the assignment of species/lineages, the alignment was trimmed to 634 bp to match the length of the COI sequences from the reference database (for NCBI GenBank Accession Numbers see Suppl. Table S1). All newly produced sequences were deposited in the NCBI GenBank under Accession Nos. PX697605–PX697840 (fresh material) and PX702236–PX702250 (historical material). The NJ tree constructed is available in newick format in the Supplementary material.

**Fig. 1:**
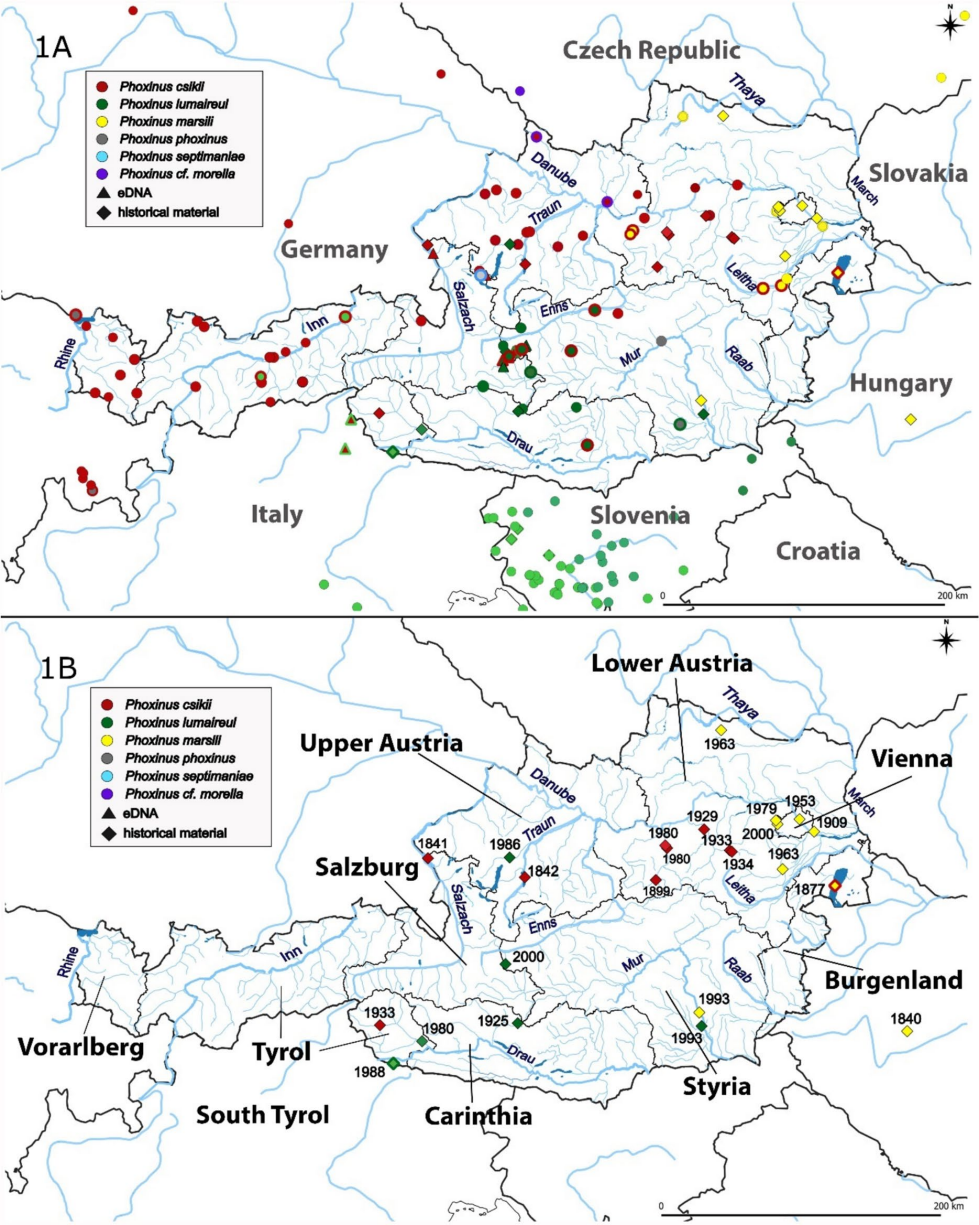
Sampling area focused on Austria and neighboring river systems. Circles represent sampling sites (fin clips or swabs), which were freshly collected. Triangles represent sampling sites where water for environmental DNA analysis was collected. Squares represent museum/historical samples. The colors represent species; the legend is given. When two species were detected at a site, two colors are given. There are several genetic lineages of *P. lumaireul*; in Austria 1a, 1c, and 1d were detected. They are denoted with different shades of green: 1a is the brightest, 1c is middle, and 1d is dark green. The major Austrian rivers are denoted. **A** All sampling sites are provided. **B** Only historical samples are marked, with their respective year of collecting

DNA samples analyzed in this study were assigned to the following genetic lineages/species: *P. csikii* (lineage 5b)*, P. lumaireul* (lineages 1a, 1c and 1d)*, P. marsilii* (lineage 9a), *P. phoxinus* (lineage 10), *P. septimaniae* (lineage 12; only detected in a state pond), and *P.* cf. *morella* (lineage 11) (Fig. 1). The latter represents the first record of this genetic lineage/putative species in Austria, and it was detected in two admixed populations in Upper Austria.

Admixed populations were also detected in Lake Constance (*P. csikii* 5b/*P. phoxinus*) in Vorarlberg. In Tirol, two admixed populations (*P. csikii* 5b*/P. lumaireul* 1a) were detected (Fig. 1). Several admixed populations (*P. csikii* 5b*/P. lumaireul* 1d) were detected in Salzburg, Styria, and Carinthia (Fig. 1). The presence of the species *P. phoxinus* was detected in two populations in Styria; one occurrence was in an admixed population together with *P. lumaireul* 1d, while the other was three specimens in Mur River, all belonging to *P. phoxinus* (Fig. 1). In Carinthia, one admixed population of *P. lumaireul* 1c/1d and one population of *P. lumaireul* 1c was detected (Fig. 1). In Lower Austria, there are several admixed populations between *P. csikii* 5b and *P. marsilii* 9a (Fig. 1).

The three DNA samples, which were extracted from specimens from state/private ponds, were identified as admixed populations of *P. phoxinus/P. septimaniae*, *P. csikii 5b/P. lumaireul* 1d, or a population of *P. lumaireul* 1d only.

**Fig. 2:**
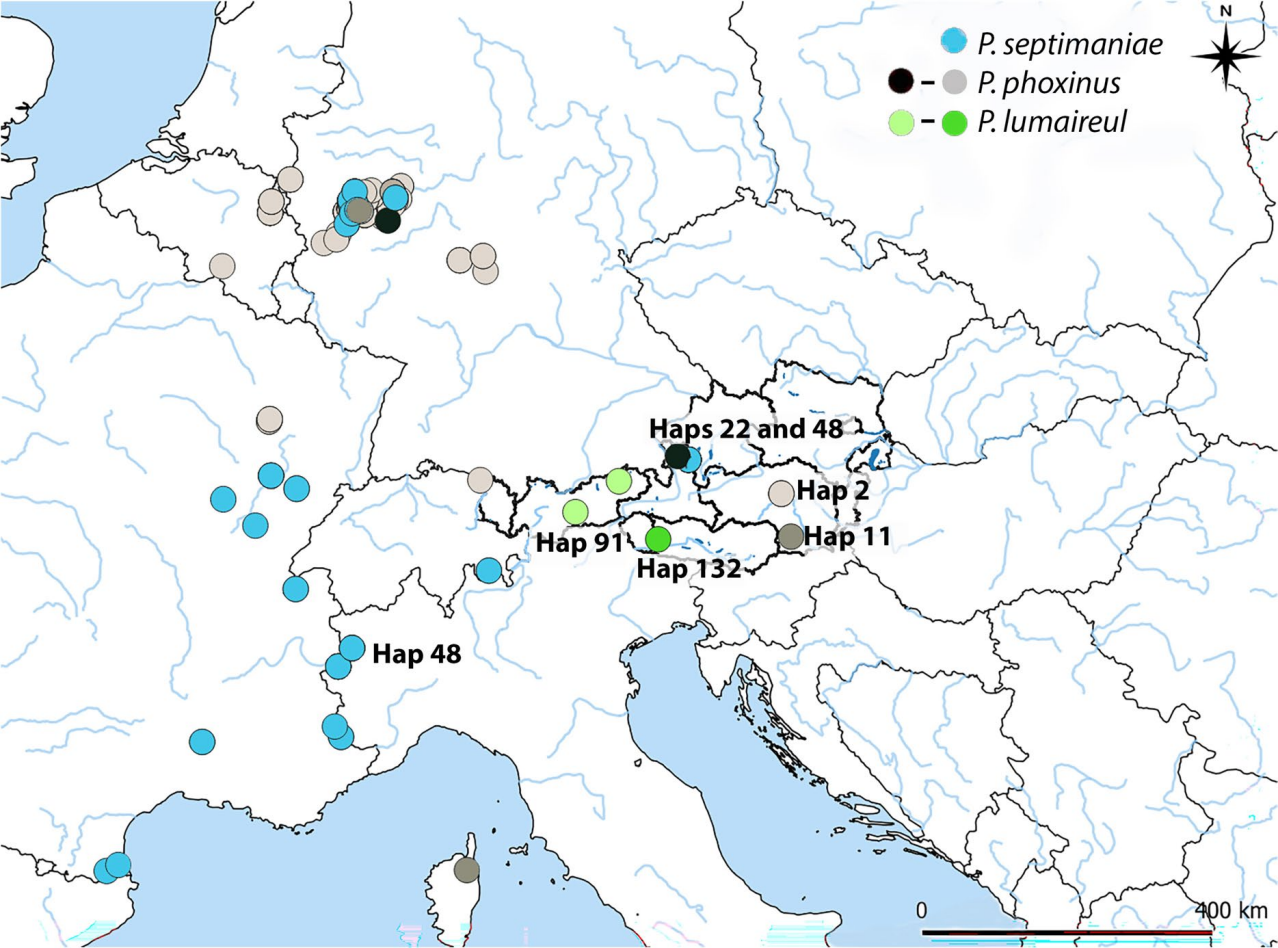
Haplotype distribution of introduced *Phoxinus* species and genetic lineages in Austria. The three *P. phoxinus* haplotypes (Haps 2, 11, and 22) are visualized with different shades of gray (light gray–black). *P. septimaniae* was detected in Austria only in a state pond and is represented by the most common haplotype of *P. septimaniae* (Hap 48, bright blue). *P. lumaireul* 1a and 1c haplotypes, Hap 91—light green and Hap 132—darker green, respectively, are unique to Austria

The ML and BI phylogenetic reconstruction (both are available in tre format in supplementary material) as well as the haplotype network reconstruction (Figure S1) were used to assess the haplotype distribution of native species/genetic lineages and to identify the possible introductory sources/routes of introduced species/lineages (haplotype distribution analysis). For the introduced haplotypes/species, there are three different haplotypes of *P. phoxinus* (lineage 10) in Austria (Suppl. Table S1). Haplotype 2 is the most common haplotype of *P. phoxinus*, spread throughout its entire species range and is found in Austria in Styria and in Lake Constance. Haplotype 11 was detected in Styria in Mur River drainage. In Germany, it is found in Sieg and Kinzing Rivers, and it was also introduced to Corsica. Haplotype 22 originates from Nister River (confluence to Sieg) in north Germany and was only identified in Austria in one of the state ponds with *Phoxinus* stock intended for renaturation projects. The three *P. phoxinus* haplotypes are visualized on Fig. 2 with different shades of gray (light gray–black). *P. septimaniae* was detected in Austria only in a state pond and is represented by the most common haplotype of *P. septimaniae*, Hap 48, which was introduced to north Germany and often co-occurs with *P. phoxinus* haplotypes. *P. lumaireul* 1a and 1c haplotypes, Hap 91 and Hap 132, respectively, are unique to Austria.

**Fig. 3:**
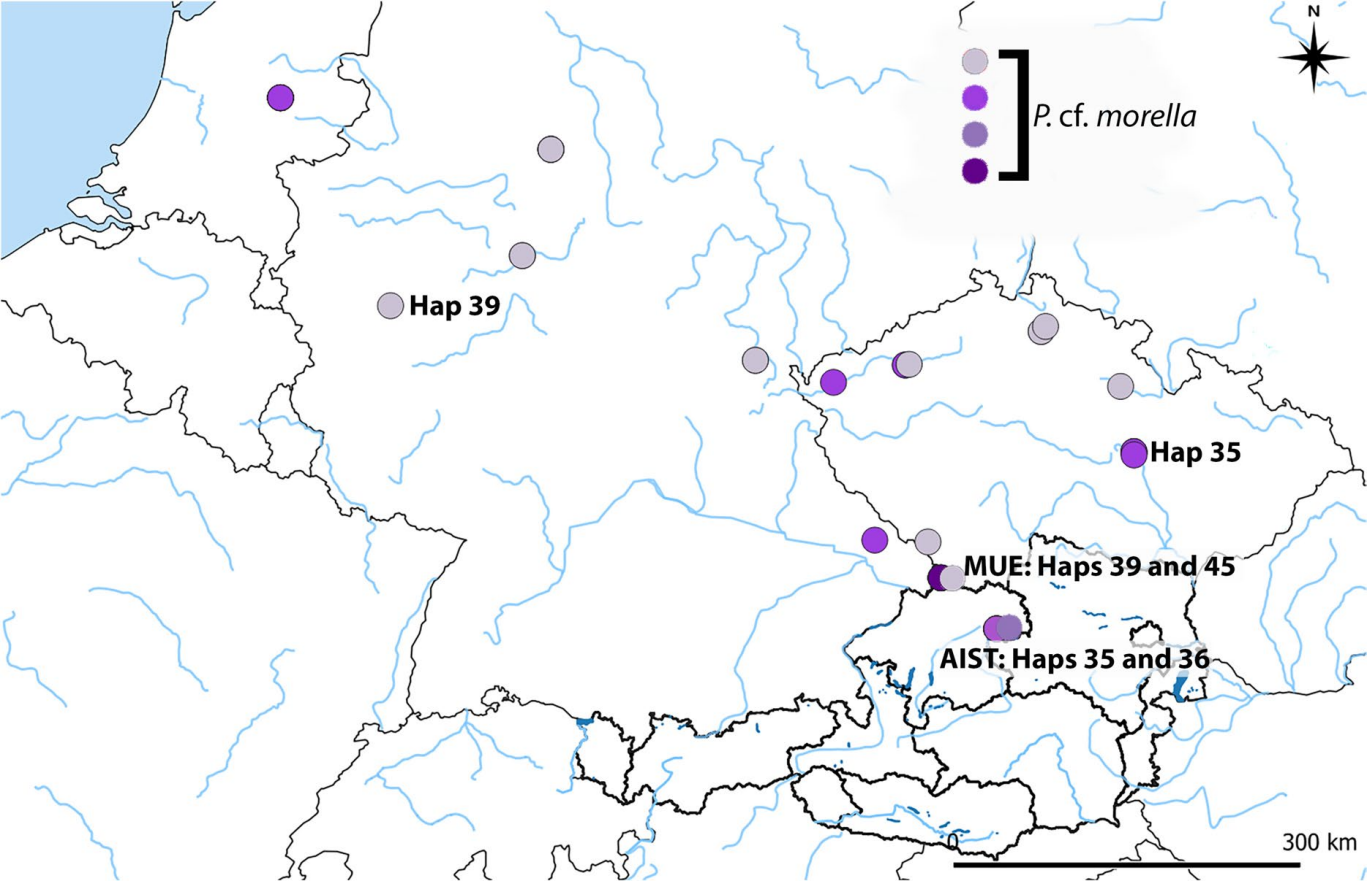
Haplotype distribution of *Phoxinus* cf. *morella*, genetic lineage 11 (coding according to Palandačić et al. (2020)). The haplotypes are visualized as different shades of violet color. Hap 36 (stream Aist) and Hap 45 (stream Große Mühl) are unique to Austria, carried by only one individual each. Haps 35 and 39 are widely spread through *P.* cf. *morella* distribution area

*P.* cf*. morella* was identified in Austria in two admixed populations (together with *P. csikii* 5b) and is represented by four different haplotypes. Hap 36 (stream Aist) and Hap 45 (stream Große Mühl) are unique haplotypes, carried by only one individual each, detected only in Austria. However, both populations also carry two widely spread haplotypes (Hap 35 and Hap 39) otherwise also present in Elbe, Wesser, and Danube drainages in Germany and Czech Republic. The haplotypes are visualized in Fig. 3 as different shades of violet color.

*P. marsilii* in Austria is represented by eight different haplotypes. Hap 232 is the most abundant haplotype, distributed across the *P. marsilii* 9a distribution range. Hap 231 is a unique haplotype carried by only one individual in a historical specimen from Neusiedler Lake. Hap 243 and Hap 248 are also unique, carried by only one specimen each, collected near Vienna in the Vienna River. This collecting site is also the most diverse, where the most abundant haplotype Hap 232, but also rare haplotypes Hap 236, Hap 246 and Hap 247 are present. The *P. marsilii* 9a haplotypes are visualized in Figure S2 in yellow, orange and brown shades.

*P. lumiareul* 1d is represented in Austria with eight different haplotypes. The most common are Hap 159 and Hap 161, distributed also in Slovenia. Hap 160 is unique and was detected in a historical specimen collected in Ager River, a confluence to Traun River in Upper Austria, while Haps 162 and 166 are also unique and were detected in Giglach Lake in Styria and Rotgülden Lake in Salzburg. Other unique haplotypes were detected in a private pond with *Phoxinus* stocked for renaturation projects (Haps 167, 168, 257). One of the most abundant haplotypes, Hap 161, is also present in this pond. The haplotypes are visualized in Figure S3, in different shades of green.

*P. csikii* 5b is the species with the widest distribution range in Austria, with 27 different haplotypes. Of those, 14 are unique, carried by only one specimen, three haplotypes are represented in two specimens each, and one haplotype is carried by three specimens (Suppl. Table S1). These haplotypes are marked gray in Figure S4. The most abundant haplotype of *P. csikii*, Hap 25 (*n* = 406), is distributed throughout Germany and Switzerland and was introduced into Italian lakes; however, in Austria, it is present only in Lake Constance. Similarly, Hap 179 (*n* = 17) is mostly distributed in Germany, and also in Western Austria. Haplotype 169 is distributed across Austria. Outside of Austria, it is only present in Bavaria, Germany. Hap 170 is mostly distributed in Western Austria, Switzerland, and Bavaria, but was also detected in Eastern Austria. Hap 173 is exclusive to Austria but not distributed in its western part. Hap 178 is distributed mostly in Agger drainage in Germany but can also be found in Lower Austria. Hap 171 is distributed in central Austria. The haplotypes are visualized in Figure S4 with red, pink and violet tones.

### eDNA metabarcoding and complete mt genome analysis

**Fig. 4:**
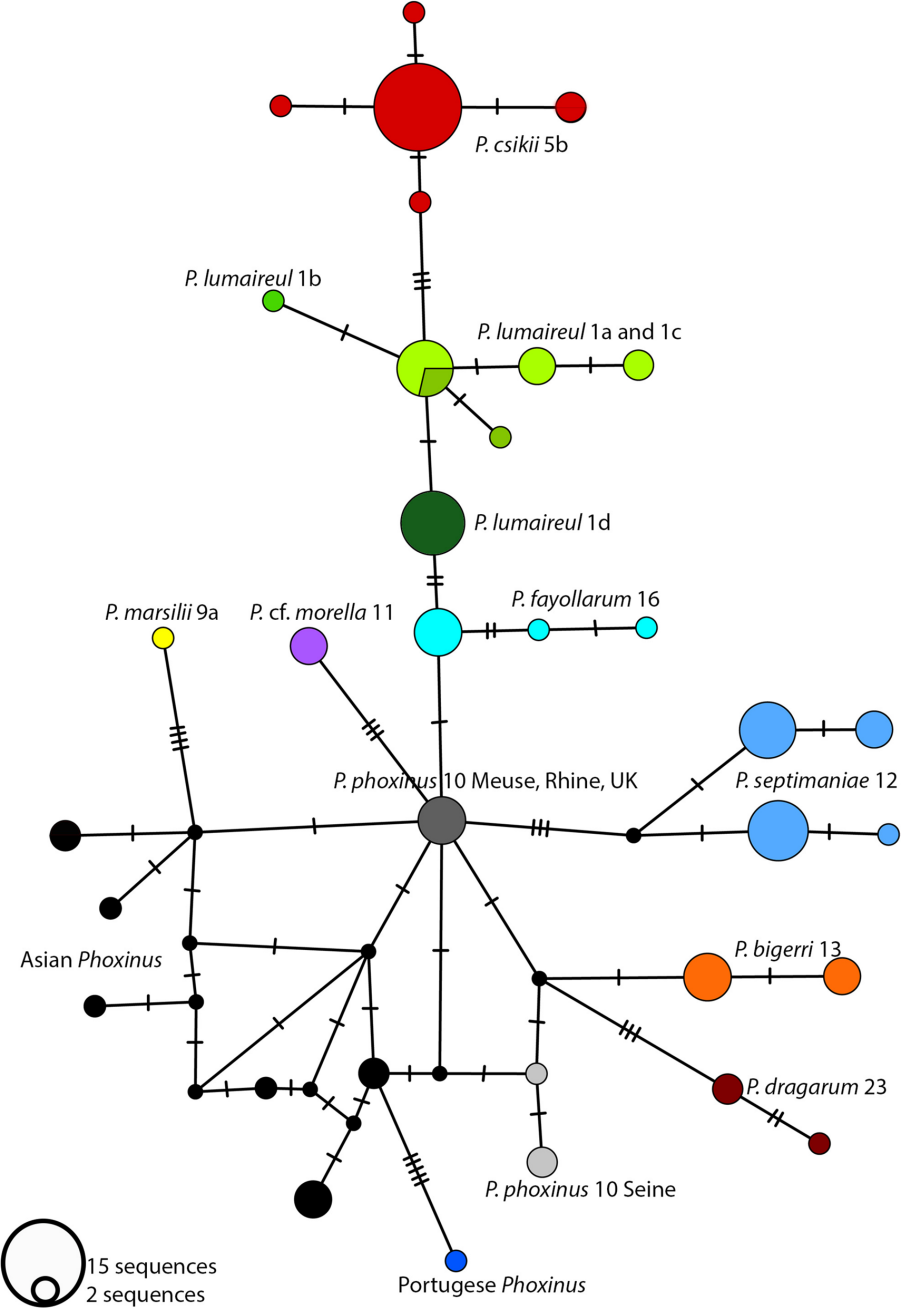
A medium-joining haplotype network was constructed using 176-bp long fragments of the mitochondrial 12S RNA gene from *Phoxinus* specimens (*N* = 104). The sequences were produced in this study using environmental DNA analysis (*N* = 18) through metabarcoding of water samples collected from various sites (see Supplementary Table S1). The samples were amplified and sequenced using MiFish-U primers (Miya et al., 2015). Additionally, sequences from the complete mitochondrial genomes of reference samples of the same length were added to the alignment (*N* = 13). Finally, all available 12S sequences from GenBank (*N* = 73) were included. Different colors represent different species and genetic lineages. Black represents different Asian *Phoxinus* species and lineages. The *P. lumaireul* 1a and 1c sequences grouped into the same haplogroup and are denoted by different shades of green: 1a is lighter green, and 1c is darker green. All sequences used are listed in Supplementary Table S1, and a phylogenetic tree constructed with these sequences is available in the supplementary material (Figure S5). The size of the circles represents the number of sequences (see the figure legend), and the lines represent the number of mutations between haplogroups. The network was constructed using PopART v1.7 software (Leigh & Bryant, 2015) with the default settings

Metabarcoding of eDNA samples (*n* = 51) collected in various alpine water bodies (Suppl. Table S1) and subsequent bioinformatic processing revealed heterogeneity in the 12S barcodes of the *Phoxinus* species complex. Based on the alignment created using 12S fragment from complete mt genomes of known genetic lineages/species, the taxonomic assignation to the species level (e.g., *P. lumaireul*) was possible. Within *P. lumaireul*, differentiation between 1a and 1c genetic lineages was impossible; however, lineage 1d formed a separate haplogroup in the haplotype network (Fig. 4) and a clade in the NJ tree (Figure S5). Of the sequences downloaded from the GenBank used in this analysis (Suppl. Table S1), *P. fayollarum* Denys et al., 2020, and *P. dragarum* Denys et al., 2020 formed distinctive haplogroups/clades; so did *P. septimaniae*. *Phoxinus phoxinus* formed two haplogroups/clades (Fig. 4, light gray and dark gray), corresponding to Seine and Meuse drainages; in the latter, two samples from the Rhine drainage and UK also clustered. The sequence of *P. cf. morella*, produced in this study, formed a haplogroup/clade with twosequences, one with locality given only as Czech Republic and the other one from Switzerland, Balerna, Breggia. In both countries, *P. cf.* *morella* is distributed. Finally, there was a sequence in GenBank, with locality “Austria.” This sequence was assigned to *P. csikii* 5b clade. All newly produced complete mitochondrial genomes were deposited in the NCBI GenBank under Accession Nos. PX873029–40, as well as 12S sequences under Accession Nos. PX707874–PX707891.

### Data collection of abiotic parameters and habitat features

Only 24% of the fresh material was sent with accompanying information, i.e., the standardized form; thus, only some basic descriptive statistics could be gathered about the sampling sites. The most *Phoxinus* (54%) were caught in lakes and 35% in streams and rivers. The rest came from private or public ponds. The substrate, where they were caught, was mostly gravel, sand, bigger stones, and a mixture of those. Most of the *Phoxinus* were caught at natural or moderately influenced sampling sites, with water plants present at about half of the sampling sites and with low or no water turbidity. They were caught at a broad range of elevation (248–2105 m a.s.l.). The descriptive statistics are available in Supplementary Table S2.

## Discussion

### Biodiversity and species distribution of Phoxinus in Austria (Aim 1)

In this study, the genetic lineage of 258 *Phoxinus* specimens was determined using the barcoding region (mt COI). The results aligned with previous findings (Palandačić et al., 2020) and confirmed the distribution of *P. marsilii* in eastern Austria (Danube, Leitha, Thaya drainages; Fig. 1), *P. lumaireul* 1d in southern Austria (Drau, Mur drainages), and *P. csikii* in central and western Austria (Rhine, Inn, Salzach, Enns, Traun, Danube drainages; Fig. 1). Besides, several admixed populations between these lineages were observed: *P. marsilii* 9a/*P. csikii* 5b and *P. csikii* 5b/*P. lumaireul* 1d*.* As these species naturally come into contact in Austria, the admixed populations may represent natural hybridization zones. The study therefore suggests that the species distributions of *Phoxinus* minnows are complex, reflecting riverine captures and historical drainage reorganization, as previously observed in *Phoxinus* (Palandačić et al., 2020; Reier et al., 2025) and other fish taxa in Austria (Zangl et al., 2020) and elsewhere (Reier et al., 2022; Vance et al., 2025). However, despite ample evidence of geological processes shaping freshwater biodiversity (see, for example, Water et al., 2026), the distributions of *Phoxinus* species in Austria are a mixture of paleohydrology and anthropogenic introductions, as discussed below.

In comparison to previous studies, the most interesting discovery was a new species record of *Phoxinus* for Austria. Namely, *P.* cf. *morella* was identified in Upper Austria in two Danube tributaries (streams Große Mühl and Aist), though only in two admixed populations together with *P. csikii* 5b. Thus, whether these admixed populations represent a natural hybridization zone is unclear, as the species *P.* cf. *morella* is naturally distributed in the neighboring Czech Republic. This finding corresponds to the observed distribution of the brook lamprey *Lampetra planeri*, a northern European species with only local occurrences in a few rivers of Upper and Lower Austria (Ratschan et al., 2021). The species is thought to have crossed the Elbe-Danube drainage boundary through stream capture (Gumpinger et al., 2008), which may also be the case for *P.* cf. *morella*. Another possible route could also be the Schwarzenberg Canal, built in the eighteenth century for drifting wood, which connected one of these two rivers (Große Mühl) with the Elbe drainage (via the Vltava River) in the Czech Republic. Natural occurrence of *P.* cf. *morella* is also supported by the haplotype distribution analysis as two unique haplotypes were detected in Austria only (Hap 36 and 45, Fig. 3). However, the occurrence of this species could also be anthropogenic, as some fish stocks for aquaculture are/were imported from the Czech Republic (personal communication with the owners of various aquaculture facilities, 2021). Thus, mitochondrial haplotype uniqueness alone does not allow discrimination between a natural origin and an introduction from currently unsampled source populations and further studies based on more specimens, sampling sites and detailed genetic analysis (such as whole genome-based hybridization analysis) are needed to determine their origin.

In previous studies (Palandačić et al., 2020), an admixed population of *P. lumaireul* 1d*/P. phoxinus* was detected in central Austria, in Styria. In this study, an additional population of *P. phoxinus* was identified in this area. The native distribution of *P. phoxinus* is in northern France, Germany, and in the Netherlands in the Rhine, Meuse, and Seine drainages (Denys et al., 2020; Palandačić et al., 2022; Sternberg et al., 2025); therefore, the populations observed in this study are considered to be introduced. A possible route of introduction is the import of *Phoxinus* from the Netherlands for garden ponds (personal communication with the owners of various aquaculture facilities, 2020); however, to confirm this information, the commercially sold *Phoxinus* should be genetically tested. The haplotype distribution analysis was not helpful for identifying possible routes of introduction, as one of the haplotypes detected in Austria (Hap 2; Fig. 2) is the most common haplotype of *P. phoxinus*, while the other (Hap 11) is also quite common and was also introduced elsewhere (e.g., in Corsica; Esposito et al. (2024)). The most common *P. phoxinus* haplotype Hap 2 was also detected in Lake Constance in an admixed population with *P. csikii* 5b. According to current studies, Lake Constance would fall within the distribution range of *P. csikii* 5b. However, according to personal communication with Dr. Julia Gaye-Siessegger (in 2023, Fisheries Research Centre Langenargen, Lake Constance), *Phoxinus* minnows were recorded for Lake Constance in historical literature (e.g., Hartmann, 1808; Rapp, 1854) but had not been caught there for decades. The first known record in Lake Constance was made in spring 2020 at the Rhine dam in Fußach Bay. The *P. phoxinus*/*P. csikii* 5b mixture is common in the Rhine drainage (De Santis et al., 2021; Esposito et al., 2024; Palandačić et al., 2022) and could be introduced as such; however, a detailed genetic analysis is needed to answer this question. Other (possible) introduced lineages in Austria are *P. lumaireul* 1a and 1c (Fig. 2). Admixed populations of *P. csikii/P. lumaireul* 1a were detected in Tyrol and in South Tyrol, with *P. csikii* being native to Tyrol and *P. lumaireul* to South Tyrol, in the Adriatic Sea drainage basin. According to word of mouth (personal observation FG), *Phoxinus* minnows were often transported as live bait between Tyrol and South Tyrol, thus between Black Sea/Adriatic Sea drainage basins, which could explain the admixed populations. The haplotype distribution analysis was not helpful in identifying the routes of introduction, as the two detected haplotypes (Haps 91 (1a lineage) and 132 (1c lineage)) were unique for Austria. This is not surprising, as there is not much information on the distribution of *Phoxinus* from South Tyrol and from Italy in general. Thus, further sampling and analysis, such as previously mentioned whole genome-based hybridization analysis, are necessary.

Although barcoding projects have generally been a quick and efficient way of revealing cryptic diversity (e.g., Feulner et al., 2006; Fontaneto et al., 2009), using mitochondrial, maternally inherited markers for species identification/assignation is problematic, particularly in the genus *Phoxinus*, where hybridization between species and genetic lineages has been demonstrated (Palandačić et al., 2015, 2017). As previously described in the introduction, the use of morphological characteristics is also inadequate for species identification (Bogutskaya et al., 2020; Palandačić et al., 2024a, 2024b; Ramler et al., 2017); due to the introductions, so is the data on species distributions (Corral‐Lou et al., 2019; De Santis et al., 2021; Reier et al., 2022). Furthermore, some nuclear genes, such as the recombination-activating gene 1 (RAG1) which is often used in the Leuciscidae family (Perea et al., 2010; Schönhuth et al., 2018), have demonstrated only limited effectiveness in species identification (Palandačić et al., 2020). To detect possible hybrid *Phoxinus* specimens, another possible nuclear region—the internal transcribed spacer 1 (ITS1)—was previously tested (Palandačić et al., 2020). ITS1 is short (350 bp in *Phoxinus*) and exists in cells in multiple copies, making it a promising candidate for amplification in historical specimens where genetic material is scarce and highly fragmented. However, ITS1 has been shown to be unsuitable for detecting hybrids within the *Phoxinus* genus due to concerted evolution and the presence of up to six paralogues (Palandačić et al., 2022). Another nuclear gene, ribosomal protein S7, was considered for a population genetic study of *Phoxinus* in the karst area and was shown to be useful for detecting hybrids. However, due to several indels, this marker proved problematic when using the Sanger sequencing method, necessitating cloning to recognize the two different alleles present in the individual (Reier et al., 2022). Although a study was conducted that proposed a microsatellite set for detecting the introgression and hybridization of *Phoxinus* species (Vucić et al., 2022), this was only tested on three *Phoxinus* species. Furthermore, when genotyped by electrophoresis, microsatellites cannot be compared between different laboratories due to the use of different machines and running conditions (e.g., Pasqualotto et al. (2007)). Consequently, the barcoding approach and/or single-marker systems were not practical or easy-to-use for detecting hybrids in *Phoxinus*, and a more exhaustive approach, such as genome-wide single nucleotide polymorphism (SNP) data analysis (Reier et al., 2025), is necessary.

### Activating citizen scientists to collect data on species distribution and for biodiversity monitoring (Aim 2)

According to Callaghan et al. (2025), citizen science has great potential for data collection and biodiversity monitoring. To protect and better manage freshwater ecosystems, community-based approaches involving regional stakeholders such as recreational fishers are highly effective (Cooke et al., 2024). In this study, both of these statements were confirmed; compared to the 20 Austrian sampling sites investigated in previous studies (Palandačić et al., 2020; Zangl et al., 2022), the current study expanded the number of sampling sites to 79 within only 2 years. In addition to time and financial constraints, collecting is also limited by the need to obtain appropriate collecting permits; in Austria, a separate permit is required for each of the nine federal states. Engaging (recreational) fishers, who usually have the necessary permits and are highly motivated to protect freshwater ecosystems, proved to be an effective and productive approach. A significant increase in the number of sites and samples collected provided a much better overview of the biodiversity and distribution of minnow species across Austria.

However, as specified in the introduction, the conservation of *Phoxinus* minnows is a multilevel process. The first step, which was to gather nationwide data on species occurrence and distribution, was accomplished with this study. The study also demonstrated that monitoring *Phoxinus* populations could be conducted with the assistance of citizen scientists, particularly fishers. It also highlighted that field biologists performing yearly surveys of freshwater habitats are valuable partners in biodiversity monitoring. Regarding the third step, renaturation, three *Phoxinus* populations from private and government ponds intended for restoration projects were analyzed within the study. Two of these populations were found to be unsuitable because they were not native to the target area. More concerning still, one of the stocks intended for renaturation projects was a mixture of *P. phoxinus* and *P. septimaniae*; the former is native to northern Germany, while the latter is native to southern France. Thus, the study demonstrated the necessity of genetic testing of fish intended for reintroduction (e.g., as in trout, Chiesa et al. (2016); Crivelli et al. (2000)), particularly in species complexes without obvious morphological differences, such as *Phoxinus*. During the project, it was also revealed that fishers, who are at the same time often local managers, are willing to test their stocks. However, there is no centralized governmental institution in Austria offering such services. In fact, management of the water bodies is fragmented, with many local managers governing small parts of water systems. Umbrella organizations, which are usually federal state fisheries associations, require information about stocking; however, they do not require information about what is being stocked or how much stock has been introduced. Some federal states require stocking with native genetic lineages only, though no genetic test is required. Consequently, renaturation projects are often initiated and led by local managers. Therefore, engaging fishers and local managers in a study such as this has a positive impact on conservation efforts. Yet, without a systematic approach, the effects are only limited.

Regarding the collection of environmental data for the conservation of *Phoxinus* minnows, the study by Sternberg et al. (2025) demonstrated the importance of such studies. They showed the differences in the ecological preferences of the two species, *P. phoxinus* and *P. csikii*, and explained their sympatric distribution in the Sieg River drainage basin (Rhine), with *P. phoxinus* preferring lower and *P. csikii* preferring higher altitudes. Such studies are crucial for understanding the impact of introduced species and their hybridization with local congeners, a phenomenon also observed in Austria (Palandačić et al., 2020). The potential of the environmental data collected by citizen scientists alongside the DNA samples is less obvious in this study due to the small amount of data gathered, which does not allow for meaningful statistical analysis. The main obstacle is the measurement of parameters such as water temperature and pH, for which appropriate equipment is required. Nevertheless, some basic information about the sampling sites was provided. For example, most samples were collected in natural and semi-natural environments, which suggests that the regulation of water systems indeed negatively affects *Phoxinus* minnow populations (Mühlbauer et al., 2016).

In conclusion, engaging recreational fishers as citizen scientists to monitor freshwater fish biodiversity has great potential to increase spatial coverage and genetic sampling, especially when less invasive collection methods, such as mucus swabs, are used. This approach also raises awareness among fishers and local managers of the potentially harmful effects of stocking. However, collecting environmental data requires proper equipment and knowledge. Consequently, citizen scientist-provided environmental data is limited and cannot be used for meaningful statistical analyses of environmental conditions, ecological studies, or habitat-genotype analyses. Since biodiversity monitoring requires standardized procedures at different levels, it cannot rely solely on data collected by citizen scientists. Nevertheless, citizen scientists can provide primary data to help scientists develop standardized biodiversity monitoring procedures.

### Using eDNA metabarcoding with MiFish primers for detecting interspecific and intraspecific genetic lineages in Austrian Phoxinus minnows

Environmental DNA metabarcoding is a powerful tool for biodiversity monitoring of fishes and a good alternative to capture-based methods (see e.g., Curto et al. (2025); and the references therein). Moreover, Valentini et al. (2016) report that the number of species detected per site using eDNA was identical to or higher than that detected using classical survey methods in 89% of cases for fishes. However, eDNA metabarcoding also has its limitations, primarily false positives and false negatives. Therefore, the detection of a certain sequence using eDNA metabarcoding does not automatically indicate the presence of a specific species; the DNA source could be contamination from wastewater (Xiong et al., 2024), for example, a false positive originating from dead bait released by anglers. Furthermore, as with barcoding using the COI gene, all the pitfalls of phylogenetic reconstruction, taxonomic implications and species identification based on one mitochondrial marker apply to eDNA metabarcoding (Funk & Omland, 2003). Thus, strict protocols for detecting contaminations, such as blanks, and rigorous filtering of acquired sequences should be applied, which was also followed in this study (see Materials and methods, 2.2). In the case of *Phoxinus*, Denys et al. (2020) report only 2–9 diagnostic sites in the 962 bp long 12S fragment used in molecular characterization of six French *Phoxinus* species. The length of the amplified 12S gene fragment commonly amplified for eDNA metabarcoding in fishes and also applied in this study is on average 172 base pairs long (herein 176 bp long; Miya et al. (2015)). Consequently, it exhibits minimal nucleotide diversity, further influenced by PCR and sequencing errors, which can lead to erroneous taxonomic identification (Ruppert et al., 2019). Nevertheless, in this study, by using the complete mitochondrial genomes of known genetic lineages as reference and by including available sequences of 12S from other studies (Suppl. Table S1), assignment of the generated 12S sequences to the species level was possible, in one case of *P. lumaireul* 1 d also to the genetic lineage level (Fig. 4). Thus, using eDNA metabarcoding with MiFish primers, which were designed for detecting species diversity, is useful for monitoring of the *Phoxinus* populations. However, to detect genetic lineages and with that possible routes of introduction, specific COI primers would be more reliable. As Valentini et al. (2016) report, sampling efforts for an eDNA metabarcoding survey are much shorter than for a classical survey: 4 h versus 3 days of sampling to achieve the same result. Classical surveys also require trained personnel for electrofishing, as well as permits for fish collection and handling. In contrast, sampling water is a quick and cost-effective process with significant potential for nationwide monitoring with the help of citizen scientists (Barbaccia et al., 2025).

## Conclusions

This study confirmed previous findings of high biodiversity of the *Phoxinus* genus in Austrian waters, which are populated by five different *Phoxinus* species. Within one of these species (*P. lumaireul*), three different genetic lineages (1a, 1c, and 1d) were detected. Of the five species, three (*P. lumaireul* 1d, *P. csikii* (lineage 5b), and *P. marsilii* (lineage 9a)) are naturally distributed here, while one (*P. phoxinus*, lineage 10) is introduced from Northern Europe, possibly from the Rhine drainage. Two of the detected genetic lineages within *P. lumaireul*, 1a and 1c, are also introduced according to this study. In addition, a fifth species, *P.* cf. *morella*, was detected in Austria, but only in two admixed populations together with *P. csikii*; further studies are needed to determine whether it occurs naturally, or it was introduced from the neighboring Czech Republic. In general, many admixed populations were detected across Austria, suggesting natural hybridization zones. Thus, as suggested in previous *Phoxinus* studies, their species distributions reflect both historical drainage reorganization and anthropogenic introductions. However, this study relied on the analysis of maternally inherited mitochondrial cytochrome oxidase I barcoding fragment, which is insufficient for hybrid detection. Previous studies have also shown that adding nuclear markers such as internal transcribed spacer 1, recombination-activating gene 1, or microsatellites provides only limited hybridization detection ability in *Phoxinus*. Therefore, genome-wide (e.g., single nucleotide polymorphism) approaches are necessary to infer hybridization zones of *Phoxinus* species in Austria and elsewhere.

In addition to documenting biodiversity and species distributions, this study made two notable methodological contributions. First, it showed that engaging citizen scientists, such as fishers, can substantially increase data collection for biodiversity and distribution studies. The citizen science approach expanded the spatial coverage of *Phoxinus* sampling sites from 20 to 79 in just 2 years. While this approach cannot replace standardized biomonitoring by professionals and provides only limited environmental data, it offers a foundation for developing standardized monitoring programs. During this study, fishers and governmental institutions also submitted DNA samples of *Phoxinus* intended for reintroduction. Analysis revealed that the genetic lineages of these stocks were often not native to the target area, highlighting the need for genetic testing of fish intended for reintroduction to prevent the unintended introduction of non-native or admixed lineages.

Second, the study demonstrated that it is possible to detect different *Phoxinus* species using eDNA analysis and the standard 12S fragment for metabarcoding. This opens opportunities for cost-effective, large-scale, and non-invasive monitoring of *Phoxinus* populations not only in Austria but potentially elsewhere. In combination with citizen science, such water sampling can be performed more easily than sampling mucous swabs or fin clips, making it accessible to a wide range of participants, from fishers to other citizen scientist groups. 

## Supplementary Information

Below is the link to the electronic supplementary material.

ESM1 **Figure S1**: A medium-joining haplotype network constructed with cytochrome oxidase I sequences of *Phoxinus* specimens from Austria from this and previous studies. COI sequences from genetic lineages, which were detected in Austria are included. Sequences from Austria are denoted with colors, while sequences from other countries are white. Lineages 1a, 1c and 1 d belong to *P. lumaireul* and are denoted with three different shades of green – light (1a) to dark (1d) green. Lineage 5b is *P. csikii* and is denoted with red. *P. septimaniae*, lineage 12, is bright blue and lineage 10 is *P. phoxinus* and is denoted with gray. Lineage 9a is *P. marsilii*, yellow and lineage 11 is *P. morella*, violet (coding according to Palandačić et al., 2020. Haplotypes of introduced species/lineages are marked. The network was constructed with PopART v1.7 software (Leigh & Bryant, 2015) using default settings (JPG 1.11 MB)

ESM2 **Figure S2**: Haplotype distribution of *P. marsilii* 9a haplotypes. They are visualized in yellow, orange and brown shades. *P. marsilii* in Austria is represented by eight different haplotypes. Hap 232 is the most abundant haplotype, distributed across the *P. marsilii* 9a distribution range. Hap 231 is an unique haplotype carried by only one individual in historical specimen from Neusiedler Lake. Hap 243 and Hap 248 are also unique, carried by only one specimen each, collected near Vienna in Vienna River. This collecting site is also the most diverse, where the most abundant haplotype Hap 232, but also rare haplotypes Hap 236, Hap 246 and Hap 247 are present (JPG 308 KB)

ESM3 **Figure S3**: Haplotype distribution of *P. lumiareul* 1 d haplotypes, eight different haplotypes were detected. The haplotypes are visualized in different shades of green. The most common are Hap 159 and Hap 161, distributed also in Slovenia. Hap 160 is unique and was detected in a historical specimen collected in Ager River, a confluence to Traun River in Upper Austria, while Haps 162 and 166 are also unique and were detected in Giglach Lake in Styria and Rotgülden Lake in Salzburg. Other unique haplotypes were detected in a private pond with *Phoxinu*s stocked for renaturation projects (Haps 167, 168, 257). One of the most abundant haplotypes, Hap 161 is also present in this pond (JPG 192 KB)

ESM4 **Figure S4**: Haplotype distribution of *P. csikii* 5b haplotypes. (1A) Unique and rare haplotypes (carried by up to three specimens) are represented in gray. (1B) For better visualization, only the haplotypes carried by more than five specimens are represented with different shades of orange, red and pink. *P. csikii* 5b is the species with the widest distribution range in Austria, with 27 different haplotypes. Of those, 14 are unique, carried by only one specimen, three haplotypes are represented in two specimens each, and one haplotype is carried by three specimens (Supplementary Table S1). The most abundant haplotype of *P. csikii,* Hap 25 (n = 406), is distributed throughout Germany, Switzerland and was introduced into Italian lakes; however, in Austria, it is present only in Lake Constance. Similarly, Hap 179 (n = 17) is mostly distributed in Germany, but also in Western Austria. Haplotype Hap 169 is distributed across Austria. Outside of Austria, it is only present in Bavaria, Germany. Hap 170 is mostly distributed in Wester Austria, Switzerland and Bavaria, but was also detected in Eastern Austria. Hap 173 is exclusive to Austria, but not distributed in its western part. Hap 178 is distributed mostly in Agger drainage in Germany, but can also be found in Lower Austria. Hap 171 is distributed in the central Austria (JPG 490 KB)

ESM5 **Figure S5**: A phylogenetic tree was reconstructed using the neighbor-joining method in MEGA 6.0 (Tamura et al., 2013), with maximum composite likelihood and 500 bootstrap replicates, based on 176-base-pair-long 12S RNA mitochondrial DNA sequences. These sequences were obtained through metabarcoding of water samples collected from various sites (see Supplementary Table S1). The samples were amplified and sequenced using MiFish-U primers (Miya et al., 2015). The sequences are coded as lineage_locality (N = 18). Additionally, sequences from the complete mitochondrial genomes of reference samples of the same length were added to the alignment; these are coded as LabID_lineage (N = 13). Finally, available sequences from GenBank were included and coded with their accession numbers (N = 73; see Supplementary Table S1). These sequences are sometimes identified as *P. phoxinus* because they originate from a time before the cryptic biodiversity of the *Phoxinus* genus was recognized. Alternatively, they originate from studies that were unaware of the changes in taxonomy; thus, the information about the locality provides more insight into the correct species identification. The reference sequences were produced using complete mitochondrial genome assembly from Illumina sequences, then cut to match the 176-bp-long eDNA fragment. Environmental DNA sequences are denoted by the full names of the sampling sites. Reference sequences are denoted with abbreviations; see Supplementary Table S1 for more information. The statistical support for the branches is below 50; thus, the bifurcations can be considered polytomies (JPG 891 KB)

ESM6 Supplementary Table S1: Information on sampling sites, specimens collected and reference sequences used in this study, which were downloaded from GenBank. A standardized form (in German), describing the sampling site, which participants/collectors were asked to fill out.(XLSX 284 KB)

ESM7 Supplementary Table S2: Information collected from the standardized forms, filled out by participants. All materials (mostly in German) prepared and used for activities with fishers and schools are also available at https://elritzen.at/. (XLSX 169 KB)

ESM8 (TXT 26.5 KB)

ESM9 (TXT 11.0 KB)

ESM10 (TXT 8.53 KB)

## Data Availability

The datasets generated during and/or analyzed during the current study are available in the GenBank under Accession Nos. PX697605–PX697840 (COI fresh material), PX702236–PX702250 (COI historical material), PX707874–PX707891 (12S) and PX873029–40 (complete mitochondrial genomes). All other relevant data are presented within the manuscript and in the supplementary material. Some problems might have occurred with uploading newick and tre formats into the system; thus, a.txt suffix was added to the file names. In order to see them in appropriate software, this suffix should be removed.
