# Supplementary figures and images for: Data collected in a citizen scientist study uncover a new species record of Phoxinus minnow for Austria
Source: Environ Monit Assess. 2026 Mar 15;198(4):319. doi: 10.1007/s10661-026-15168-6 (PMC12989464; doi:10.1007/s10661-026-15168-6)

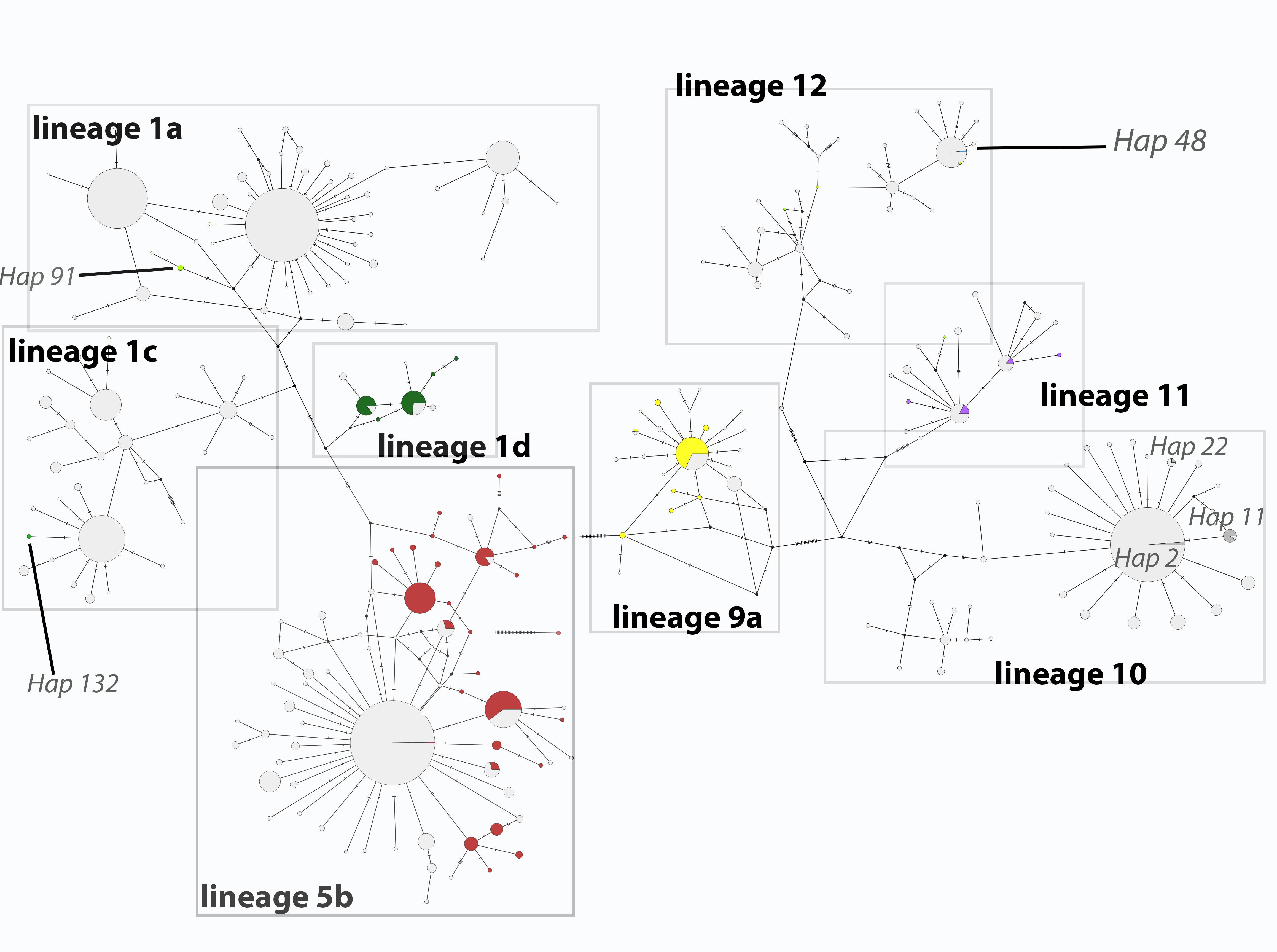

Supplement: Supplementary file 1 — Figure S1: A medium-joining haplotype network constructed with cytochrome oxidase I sequences of Phoxinus specimens from Austria from this and previous studies. COI sequences from genetic lineages, which were detected in Austria are included. Sequences from Austria are denoted with colors, while sequences from other countries are white. Lineages 1a, 1c and 1 d belong to P. lumaireul and are denoted with three different shades of green – light (1a) to dark (1d) green. Lineage 5b is P. csikii and is denoted with red. P. septimaniae, lineage 12, is bright blue and lineage 10 is P. phoxinus and is denoted with gray. Lineage 9a is P. marsilii, yellow and lineage 11 is P. morella, violet (coding according to Palandačić et al., 2020. Haplotypes of introduced species/lineages are marked. The network was constructed with PopART v1.7 software (Leigh & Bryant, 2015) using default settings (JPG 1.11 MB) [file 10661_2026_15168_MOESM1_ESM.jpg]

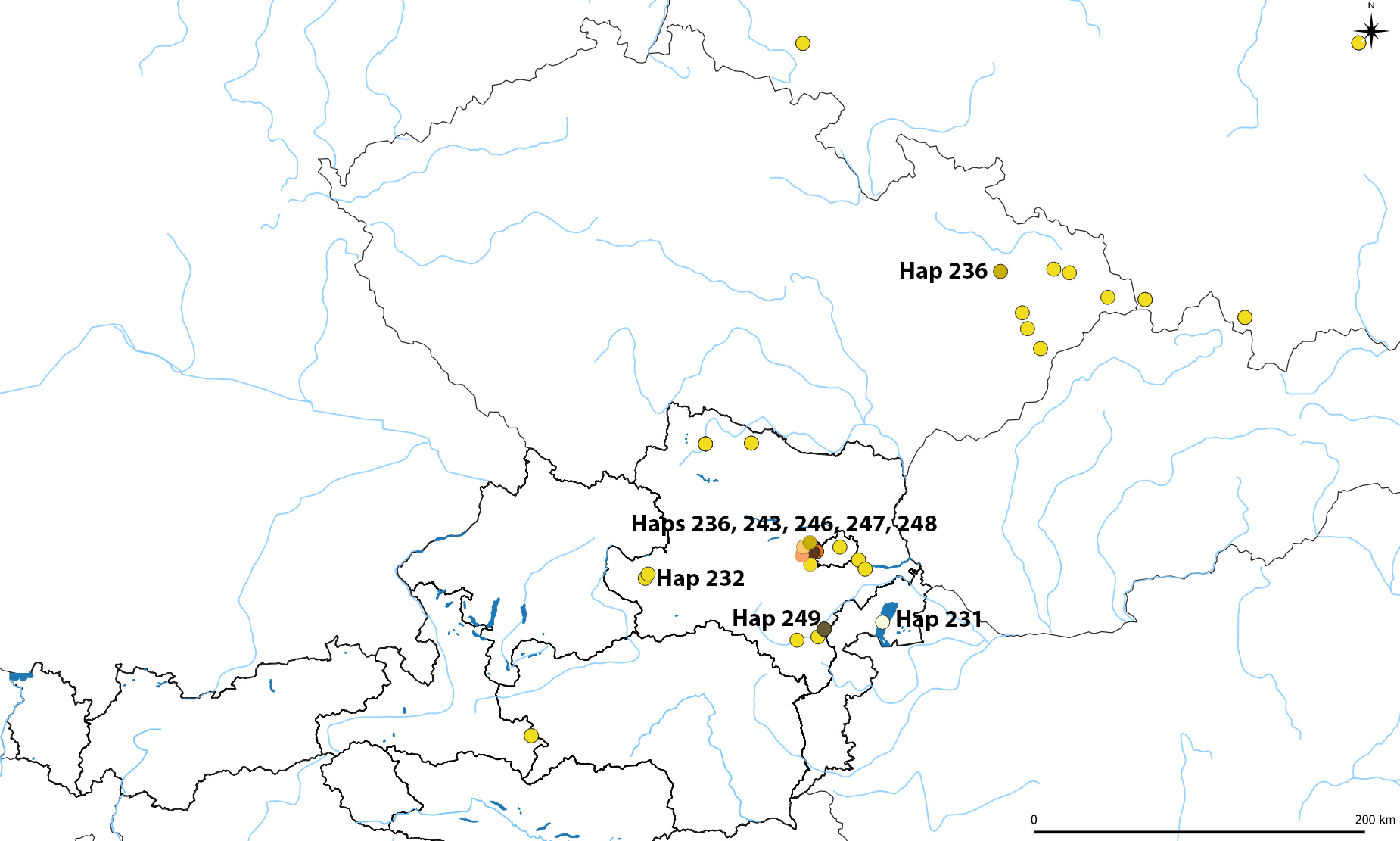

Supplement: Supplementary file 2 — Figure S2: Haplotype distribution of P. marsilii 9a haplotypes. They are visualized in yellow, orange and brown shades. P. marsilii in Austria is represented by eight different haplotypes. Hap 232 is the most abundant haplotype, distributed across the P. marsilii 9a distribution range. Hap 231 is an unique haplotype carried by only one individual in historical specimen from Neusiedler Lake. Hap 243 and Hap 248 are also unique, carried by only one specimen each, collected near Vienna in Vienna River. This collecting site is also the most diverse, where the most abundant haplotype Hap 232, but also rare haplotypes Hap 236, Hap 246 and Hap 247 are present (JPG 308 KB) [file 10661_2026_15168_MOESM2_ESM.jpg]

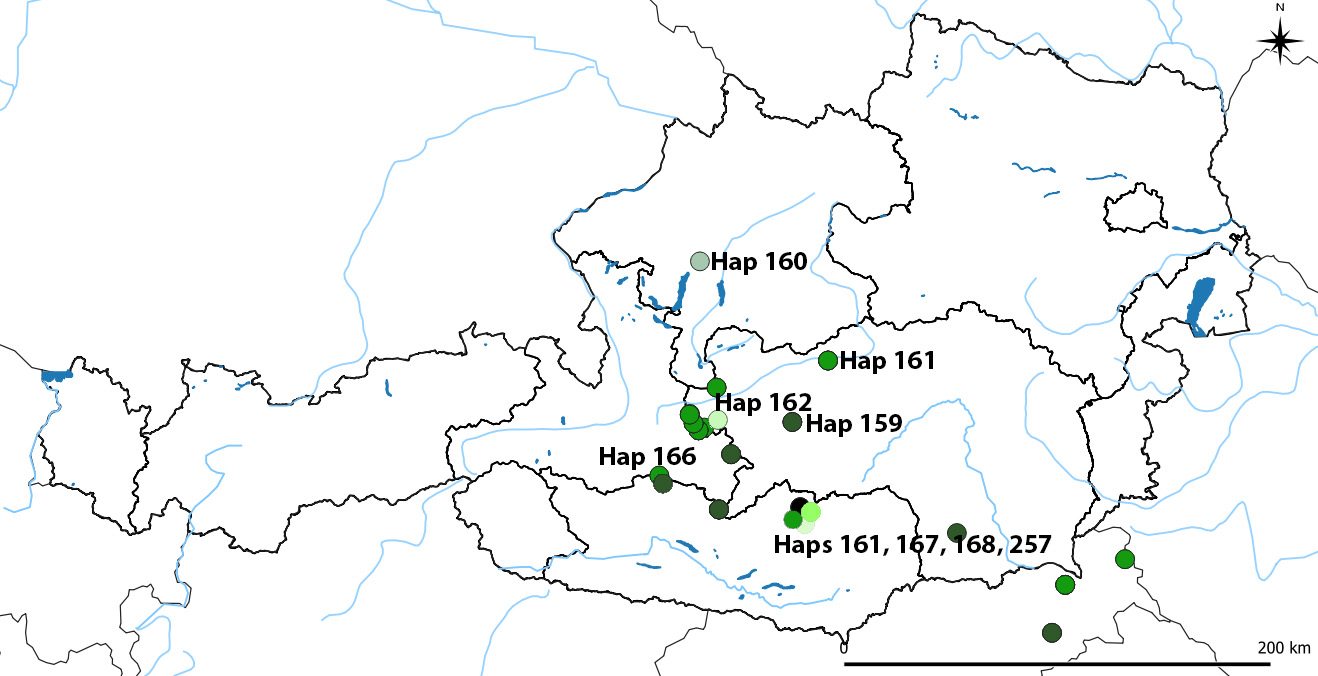

Supplement: Supplementary file 3 — Figure S3: Haplotype distribution of P. lumiareul 1 d haplotypes, eight different haplotypes were detected. The haplotypes are visualized in different shades of green. The most common are Hap 159 and Hap 161, distributed also in Slovenia. Hap 160 is unique and was detected in a historical specimen collected in Ager River, a confluence to Traun River in Upper Austria, while Haps 162 and 166 are also unique and were detected in Giglach Lake in Styria and Rotgülden Lake in Salzburg. Other unique haplotypes were detected in a private pond with Phoxinus stocked for renaturation projects (Haps 167, 168, 257). One of the most abundant haplotypes, Hap 161 is also present in this pond (JPG 192 KB) [file 10661_2026_15168_MOESM3_ESM.jpg]

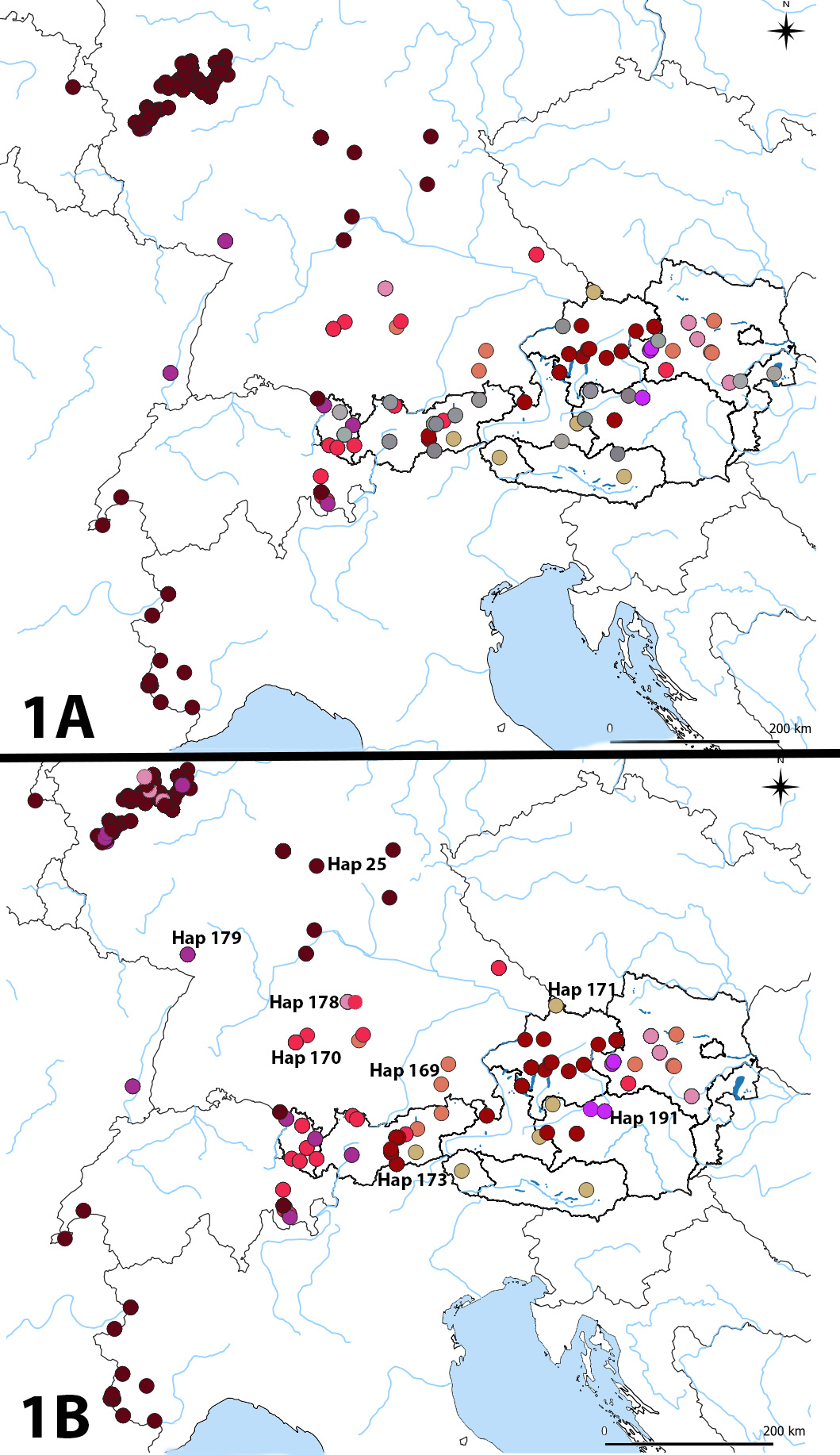

Supplement: Supplementary file 4 — Figure S4: Haplotype distribution of P. csikii 5b haplotypes. (1A) Unique and rare haplotypes (carried by up to three specimens) are represented in gray. (1B) For better visualization, only the haplotypes carried by more than five specimens are represented with different shades of orange, red and pink. P. csikii 5b is the species with the widest distribution range in Austria, with 27 different haplotypes. Of those, 14 are unique, carried by only one specimen, three haplotypes are represented in two specimens each, and one haplotype is carried by three specimens (Supplementary Table S1). The most abundant haplotype of P. csikii, Hap 25 (n = 406), is distributed throughout Germany, Switzerland and was introduced into Italian lakes; however, in Austria, it is present only in Lake Constance. Similarly, Hap 179 (n = 17) is mostly distributed in Germany, but also in Western Austria. Haplotype Hap 169 is distributed across Austria. Outside of Austria, it is only present in Bavaria, Germany. Hap 170 is mostly distributed in Wester Austria, Switzerland and Bavaria, but was also detected in Eastern Austria. Hap 173 is exclusive to Austria, but not distributed in its western part. Hap 178 is distributed mostly in Agger drainage in Germany, but can also be found in Lower Austria. Hap 171 is distributed in the central Austria (JPG 490 KB) [file 10661_2026_15168_MOESM4_ESM.jpg]

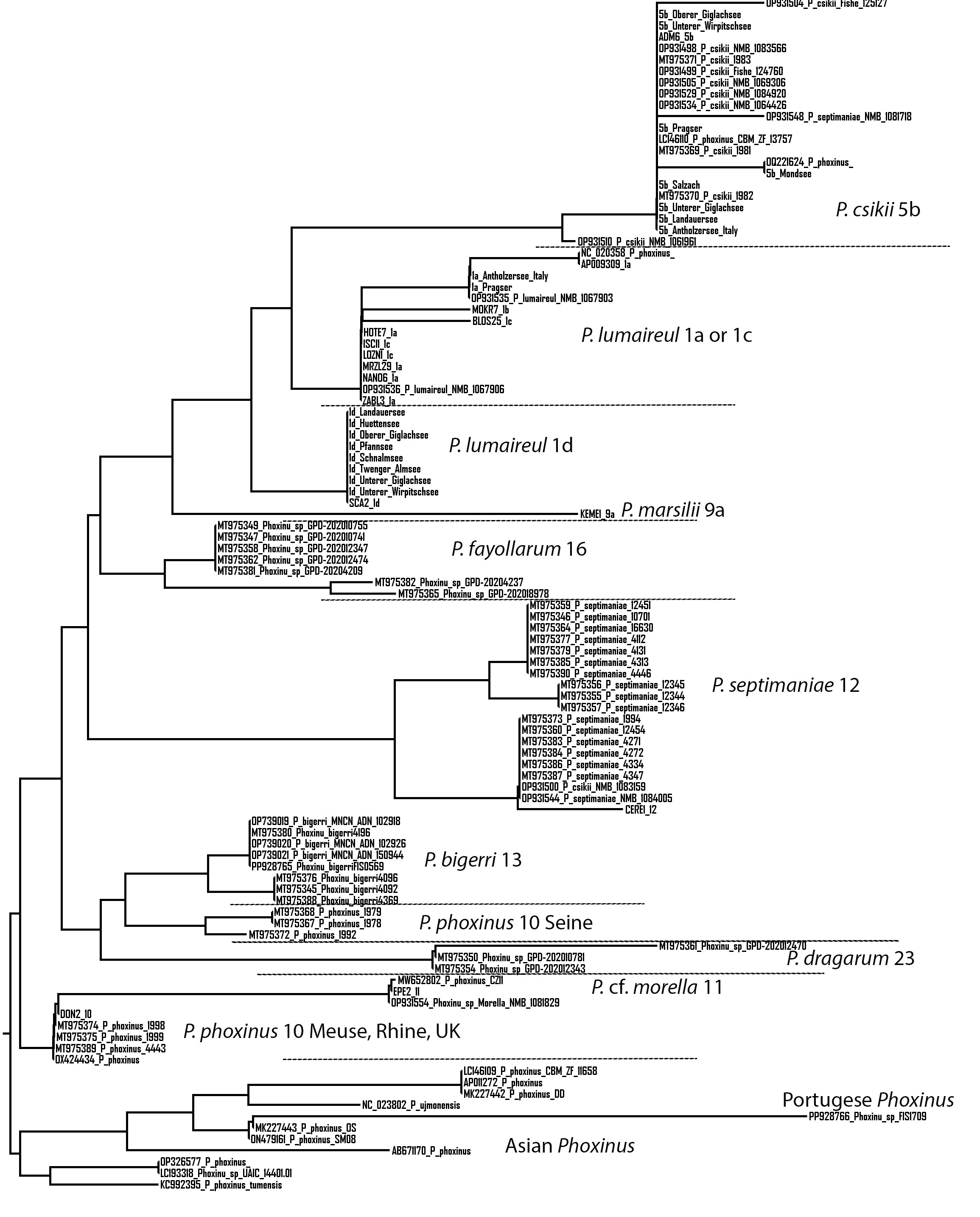

Supplement: Supplementary file 5 — Figure S5: A phylogenetic tree was reconstructed using the neighbor-joining method in MEGA 6.0 (Tamura et al., 2013), with maximum composite likelihood and 500 bootstrap replicates, based on 176-base-pair-long 12S RNA mitochondrial DNA sequences. These sequences were obtained through metabarcoding of water samples collected from various sites (see Supplementary Table S1). The samples were amplified and sequenced using MiFish-U primers (Miya et al., 2015). The sequences are coded as lineage_locality (N = 18). Additionally, sequences from the complete mitochondrial genomes of reference samples of the same length were added to the alignment; these are coded as LabID_lineage (N = 13). Finally, available sequences from GenBank were included and coded with their accession numbers (N = 73; see Supplementary Table S1). These sequences are sometimes identified as P. phoxinus because they originate from a time before the cryptic biodiversity of the Phoxinus genus was recognized. Alternatively, they originate from studies that were unaware of the changes in taxonomy; thus, the information about the locality provides more insight into the correct species identification. The reference sequences were produced using complete mitochondrial genome assembly from Illumina sequences, then cut to match the 176-bp-long eDNA fragment. Environmental DNA sequences are denoted by the full names of the sampling sites. Reference sequences are denoted with abbreviations; see Supplementary Table S1 for more information. The statistical support for the branches is below 50; thus, the bifurcations can be considered polytomies (JPG 891 KB) [file 10661_2026_15168_MOESM5_ESM.jpg]
